# The role of the intratumoral microbiota in breast cancer metastasis and immune regulation: mechanisms and therapeutic implications

**DOI:** 10.3389/fimmu.2026.1840903

**Published:** 2026-05-29

**Authors:** Feifan Leng, Ruiying Xia, Jianying Pei, Yuzhe Shi, Chenjie Gao, Fang Wang, Yan Li

**Affiliations:** 1 School of Life Science and Engineering, Lanzhou University of Technology, Lanzhou, Gansu, China; 2 Department of Clinical Laboratory Center, Gansu Provincial Maternity and Child-Care Hospital, Lanzhou, Gansu, China; 3 Department of Biochemistry and Molecular Biology, Medical College of Northwest Minzu University, Lanzhou, Gansu, China

**Keywords:** intratumoral microbiota, breast cancer, metastasis, tumor immune microenvironment, immune regulation, immune checkpoints

## Abstract

Breast cancer metastasis remains the leading cause of cancer-related mortality and is closely linked to immune evasion and tumor microenvironment (TME) remodeling. Emerging evidence suggests that intratumoral microbiota (ITM), typically low in biomass and predominantly intracellular, may be associated with tumor progression. This review summarizes the compositional features of ITM and their potential roles in metastasis, including epithelial–mesenchymal transition (EMT), circulating tumor cell (CTC) survival, pre-metastatic niche formation, and distant colonization. Mechanistically, ITM may influence these processes through immune-related signaling pathways (e.g., PRR-mediated cascades) and modulation of immune cell function. However, current evidence is largely derived from preclinical or correlative studies, and causal roles in human breast cancer remain unproven. Methodological challenges associated with low biomass further complicate interpretation. We also discuss microbiota-targeted strategies, including probiotics, antibiotics, and fecal microbiota transplantation, which remain experimental. Future studies using rigorous methodologies and longitudinal human data are required to clarify the role of ITM and its clinical potential.

## Graphical Abstract

**Figure:**
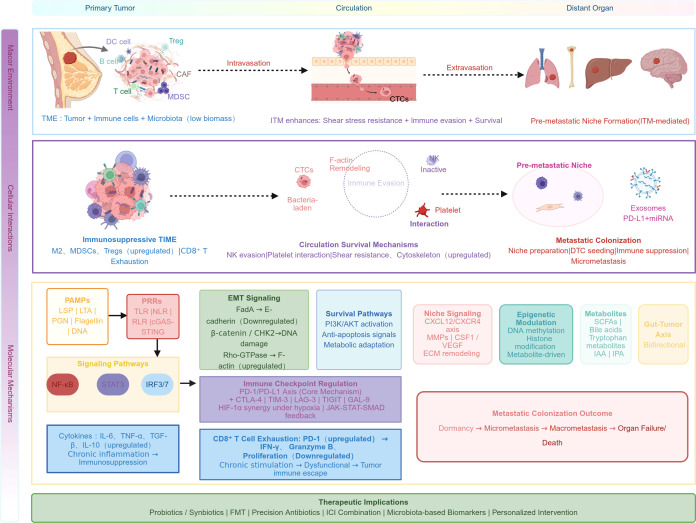
Potential mechanistic landscape of intratumoral microbiota in breast cancer metastasis This schematic summarizes the hypothesized multi-axis interactions between the intratumoral microbiome (ITM) and the host immune-metastatic cascade. Given the low-biomass nature of the tumor microbiome and associated technical challenges, the proposed pathways reflect varying strengths of evidence: compositional features and spatial co-localization with immunosuppressive cells are supported by human clinical observations. In contrast, the specific roles of ITM in promoting circulating tumor cell (CTC) survival—such as resistance to shear stress—and in pre-metastatic niche formation are derived primarily from preclinical animal models and *in vitro* assays. Accordingly, the molecular signaling pathways and therapeutic intervention strategies illustrated in the lower panel (e.g., fecal microbiota transplantation [FMT] and precise antibiotic regimens) should be interpreted as conceptual, hypothesis-generating frameworks for future research, rather than as established causal mechanisms or validated clinical protocols in human breast cancer. Created in https://BioRender.com.

## 1 Introduction

According to the International Agency for Research on Cancer (IARC), breast cancer is one of the most prevalent malignancies among women worldwide, accounting for approximately 11.6% of newly diagnosed cancers and 6.9% of global cancer-related deaths (1). Distant metastasis represents the leading cause of mortality in breast cancer patients, with approximately 20%–30% of individuals developing metastatic lesions during disease progression (2, 3). Despite advances in targeted therapy and immunotherapy in recent decades, clinical treatment options for metastatic breast cancer remain limited. Immune evasion is widely considered a critical process in breast cancer metastasis, involving complex interactions among tumor cells, immune cells, and the TME (4, 5). This process is linked to dysregulated coordination between innate and adaptive immunity, as well as the functional exhaustion of effector immune cells, which results from aberrant activation of the immune checkpoint pathway (6).

The TME is increasingly recognized as a complex ecosystem comprising tumor cells, immune cells, stromal cells, and various non-cellular components (7). Emerging evidence suggests that microbiota may reside within breast tumor tissues. Compared with other body sites, intratumoral microbiota are characterized by low biomass and predominantly intracellular localization. Given these features, careful methodological considerations are required (8, 9). These microbiota may establish persistent residence within tumor tissue and exhibit relatively conserved intracellular features, although their composition and abundance appear to be influenced by tumor type, anatomical location, and host-specific factors (10). Moreover, they have been reported to be enriched in clusters of CTCs, where they may be associated with altered immune responses and metastatic potential (11).

Current studies suggest that ITM may be involved in the regulation of tumor-related processes through multiple mechanisms, including modulation of inflammatory responses, remodeling of the immune microenvironment, and alterations in metabolic pathways (12, 13). Due to their intracellular localization, these microbiota may persist within the TME and potentially contribute to breast cancer metastasis by affecting immune cell function, inflammatory signaling, and cytoskeletal dynamics (14). For example, preclinical studies suggest that certain microbiota taxa may influence immune cell behavior, such as macrophage polarization and T-cell activation, and affect tumor cell proliferation and migration. These findings require further investigation in human models. Additionally, microbiota metabolites, including short-chain fatty acids and lipopolysaccharides, have been proposed to modulate local immune responses (15). However, these observations are primarily derived from animal models and *in vitro* experiments, and their applicability to human breast cancer remains unproven.

This review aims to summarize the compositional characteristics and potential functional roles of ITM in breast cancer, with a particular focus on their associations with metastasis and immune regulation, and to critically evaluate microbiota-based therapeutic strategies (16). Additionally, the methodological limitations inherent to low-biomass microbiome studies are highlighted to provide a rigorous scientific framework and guidance for future research.

## 2 Biological mechanisms of breast cancer metastasis

**Figure 1:**
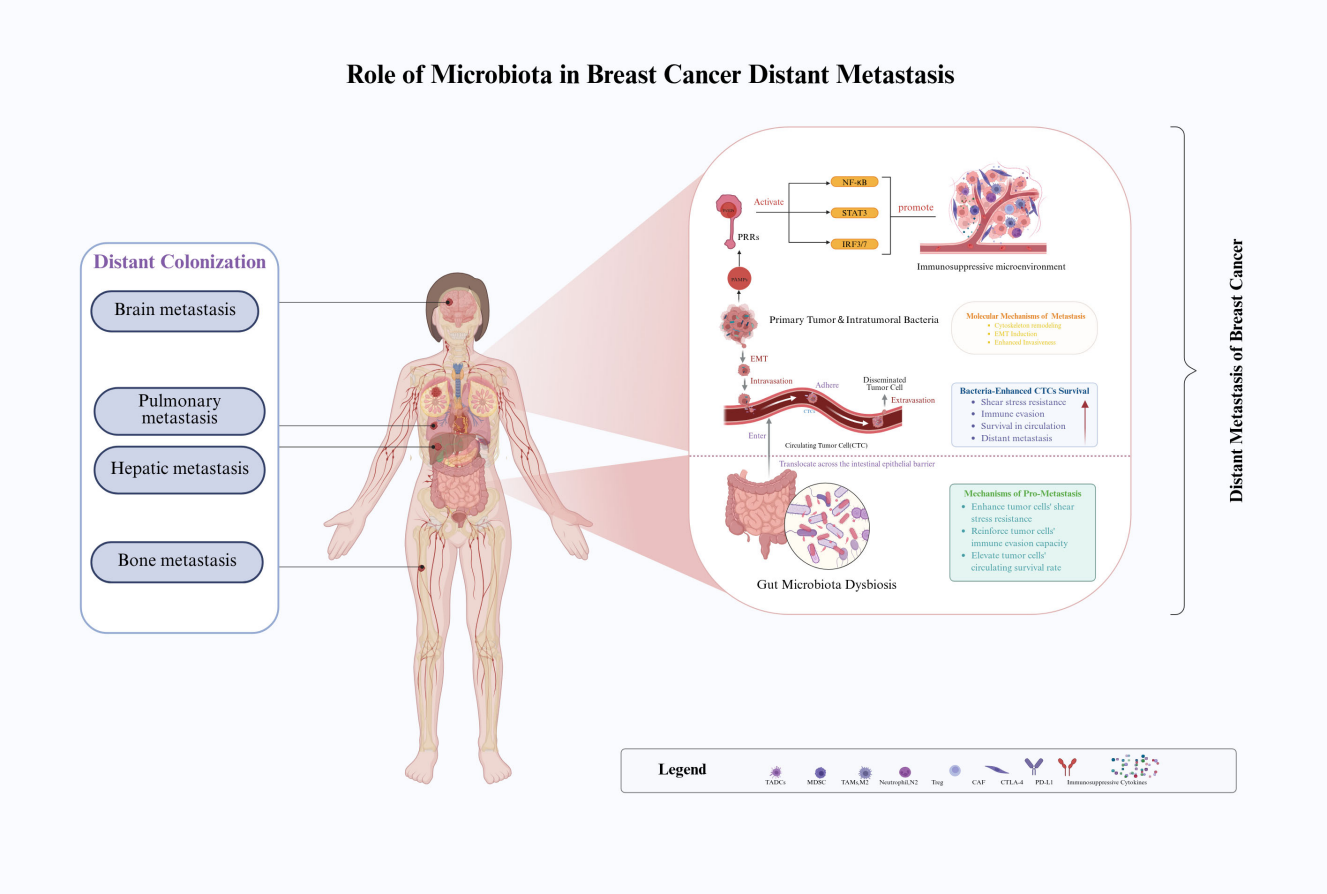
Role of microbiota in breast cancer distant metastasis: a multi-axis mechanistic view. This schematic summarizes the hypothesized roles of intratumoral and gut microbiota dysbiosis in breast cancer metastasis based on preclinical models and correlative human studies. While clinical observations have identified microbial signals in metastatic organs, the specific mechanistic interactions—such as the role of intratumoral microbiota (ITM) in enhancing shear stress resistance and circulating tumor cell (CTC) survival—are primarily derived from breast cancer-specific animal models (e.g., MMTV-PyMT) and *in vitro* assays. The depicted processes of epithelial-to-mesenchymal transition (EMT), immune evasion, and pre-metastatic niche formation represent conceptual biological frameworks that require further validation to establish definitive clinical causality in human cohorts. Red arrows indicate enhancement of biological processes. Created in https://BioRender.com.

Metastasis in breast cancer is a multistep, multistage process that generally encompasses local invasion, entry into the circulatory system, and colonization at distant sites (3) (**Figure 1**). During the local invasion phase, primary tumor cells undergo epithelial–mesenchymal transition (EMT), losing epithelial polarity and adhesive properties while acquiring mesenchymal-like migratory and invasive capabilities (17, 18). This process has been extensively documented in *in vitro* studies and animal models. Subsequently, tumor cells invade adjacent tissues either as single cells or clusters and intravasate through blood or lymphatic vessel walls, forming CTCs (19). During hematogenous dissemination, CTCs must withstand shear forces and evade immune clearance. Evidence from some mouse models indicates that a small fraction of CTCs can survive in circulation via mechanisms such as cytoskeletal remodeling (11), activation of anti-apoptotic pathways, or the formation of protective complexes with platelets (20–22), potentially enabling colonization of distant organs. In addition to hematogenous spread, lymphatic metastasis represents another early dissemination route (23), largely dependent on tumor cell invasiveness of the lymphatic endothelium and the expression of chemokine receptors such as CCR7 (24). Clinical observations suggest that early lymph node metastasis in breast cancer is associated with these mechanisms. Ultimately, disseminated tumor cells may colonize distant organs such as the lung, liver, bone, or brain, entering a dormant phase before possible reactivation and the formation of macroscopic metastases (25). Successful colonization may be influenced by tumor cell adaptation to the pre-metastatic niche, as well as interactions with local immune components, metabolic factors, and microbiota (26–28).

Recent studies suggest that gut microbiota may modulate immune responses and potentially enhance immunotherapy efficacy by influencing interactions within the tumor immune microenvironment (29). In addition, preclinical studies indicate that microbiota may influence immune signaling, cytoskeletal organization, and tumor cell survival. However, their relevance in human breast cancer remains unclear. For instance, in the MMTV-PyMT mouse model, bacteria associated with CTCs showed enhanced tumor cell survival under mechanical stress (11). Certain microbiota taxa, including *Fusobacterium nucleatum* and *Staphylococcus epidermidis*, have been reported to modulate local inflammatory signaling and immune checkpoint molecule expression (30–32).

In summary, breast cancer metastasis involves intricate interactions among tumor-intrinsic features, host immune regulation, and microenvironmental factors. ITM may be associated with multiple steps in this process, but causal relationships in humans have not been demonstrated. Future studies employing rigorous methodological controls and longitudinal human samples are necessary to clarify the roles and mechanisms of ITM in breast cancer metastasis and to evaluate their potential as targets for intervention.

## 3 Role of intratumoral microbiota

### 3.1 Compositional features of the intratumoral microbiota

Recent studies have identified low-abundance microbiota signals in breast tumor tissues (33), with differences observed between tumor and normal tissues. For instance, some bacterial taxa, including *Bacillus* and *Staphylococcus*, are reported to be enriched in tumor tissues, whereas *Prevotella* and *Lactococcus* are more prevalent in normal tissues (34, 35). Furthermore, fungal species (e.g., *Candida*), viruses, and mycoplasmas have also been observed in some studies (36–38). These observations are primarily derived from 16S rRNA or metagenomic sequencing, complemented in some cases by fluorescence *in situ* hybridization or spatial transcriptomics for partial validation. These findings are descriptive, and causal relationships with tumor development or progression remain to be established.

Several studies indicate that microbiota diversity is generally reduced in breast cancer tissues compared with normal tissues, characterized by decreased dominance of commensal taxa and a relative increase in potential pathogenic bacteria. In high-grade malignancies, one study reported a decrease in *Bacteroidaceae* and an increase in *Agrococcus*, accompanied by upregulation of pathways related to biotin synthesis and glycerophospholipid metabolism (39). These metabolic changes may reflect reprogramming of the TME, but it remains unclear whether they are driven by microbiota or are secondary effects of tumor progression. Furthermore, microbiota composition may also vary with tumor subtype and stage. However, these observations are primarily descriptive and do not establish causal relationships (40). For example, microbiota dysbiosis in male patients tends to be localized to the tumor site, whereas in female patients, broader tissue-level differences are observed (41). Across molecular subtypes, triple-negative breast cancer (TNBC) has been associated with enrichment of *Ralstonia* and *Fusobacterium*, whereas HER2-positive tumors more commonly harbor *Streptococcus* and *Bosea* (42, 43). These findings suggest potential subtype specificity of ITM (44).

While microbiota-based therapies may enhance breast cancer treatment, their application is still in the early stages. Clinically, patients with microbiota enriched in probiotic-like taxa, such as *Lactococcus*, had longer disease-free survival (DFS), whereas enrichment of *Fusobacterium* was associated with poorer prognosis in certain studies (45, 46). These observations are primarily correlative and cannot yet be applied as predictive clinical markers. Viral nucleic acids, including HPV and EBV, have been detected in some breast cancer tissues, with higher detection rates in TNBC and HR^+^ subtypes (47, 48). While these findings suggest a potential role of viral infection in tumor-related processes, their pathogenic significance remains controversial.

Beyond compositional differences, ITM exhibits spatial and temporal heterogeneity (49, 50). Spatially, *Fusobacterium nucleatum* tends to localize at the invasive front of tumors (51), whereas *Haemophilus influenzae* is more frequently found in the tumor stroma. These distributions may reflect local microenvironmental conditions, but their functional significance remains unclear. Fungal-enriched regions have been associated with elevated lactate levels, indicating that microbiota metabolism may influence local immune or metabolic environments (52). Temporally, some studies have reported a decrease in *Bacteroidia* and a relative increase in *Agrococcus* abundance during tumor progression (39). These changes may reflect selective pressures imposed by the evolving TME, although whether they contribute to tumor progression or metastasis remains to be determined.

### 3.2 Methodological challenges and validation strategies in intratumoral microbiota research

Microbiota signals in breast tumor tissues are typically of low biomass, making them highly susceptible to contamination and technical biases that can compromise data authenticity and mechanistic interpretation. Therefore, stringent sterile procedures during sample collection are essential to minimizing contamination from skin or environmental microbiota. Commonly employed approaches include needle biopsies, surgical resection specimens, and frozen tissues. Negative controls—such as no-template controls (NTCs), environmental blank controls (EBCs), and reagent blanks—should be incorporated to assess potential contamination from reagents (“kitome”) and cross-sample contamination (“splashome”). Sample processing and nucleic acid extraction should include host-read depletion and decontamination algorithms, although differences in analytical strategies can still affect data interpretation. To address these challenges, recent studies have improved sensitivity and reliability in low-biomass samples by optimizing nucleic acid extraction and amplification protocols, combining absolute quantification methods (e.g., qPCR), and employing enhanced 16S library preparation strategies, including targeted fragment enrichment (11). Experimental designs should include multi-batch replication, with batch information recorded to allow appropriate data correction.

Bioinformatic decontamination algorithms are essential for identifying and filtering false signals originating from reagents or the environment. When interpreting data, researchers should clearly define the scope and limitations of each dataset. Recent studies indicate that the PRISM framework, which removes technical artifacts and applies a gradient-boosted tree (XGBoost) model, can effectively enhance the reliability of microbiota detection (53). To further validate the presence and potential functions of microbiota, orthogonal verification methods are recommended. Fluorescence *in situ* hybridization (FISH) and spatial transcriptomics can confirm microbiota distribution and intracellular localization, whereas transmission electron microscopy provides direct structural evidence. Some studies additionally utilize selective antibiotic treatments or cell separation experiments to distinguish intracellular from extracellular microbiota and assess potential biological functions. For instance, researchers have systematically analyzed ITM across multiple solid tumors using rigorous contamination control, combined with FISH, electron microscopy, and culture-based methods, supporting the potential existence of intracellular microbiota within tumor tissues. Nevertheless, even with multimodal verification, results in a low-biomass context must be interpreted cautiously and require replication across independent studies.

Different types of microbiota signals have distinct biological implications. DNA sequencing primarily reflects the presence of microbiota genetic material and does not necessarily indicate viability or activity. RNA signals more closely represent transcriptionally active microbiota, whereas culture-based evidence indicates the presence of potentially viable bacteria. Therefore, functional interpretations should integrate DNA, RNA, and culture data and clearly distinguish between preclinical animal models, *in vitro* experiments, and human tissue samples to avoid conflating mere presence with functional relevance.

In summary, the low-biomass nature of ITM necessitates stringent standards in experimental design, data processing, and result validation. Future studies should consider establishing tabular or scoring frameworks to comprehensively present evidence linking specific microbiota, pathways, and phenotypes, stratified by level of evidence (preclinical, human correlative, and mechanistic inference). Through such systematic methodological frameworks, researchers can more reliably identify microbiota signals in low-biomass tumors, interpret their potential functions appropriately, and lay a solid foundation for subsequent mechanistic studies and clinical translation. Any investigation into their roles in tumor development and metastasis should proceed with rigorous evaluation based on these principles.

### 3.3 Intratumoral microbiota and tumor development

Whether ITM acts as an active regulator in breast cancer tumor tissue remains controversial. Evidence suggests that ITM may interact with the TME, potentially influencing signaling pathways, metabolism, and treatment responses, rather than acting as independent drivers. Most supporting data come from *in vitro* studies or animal models (**Table 1**). At the level of tumor proliferation, certain microbiota taxa, such as *Fusobacterium nucleatum*, have been reported to affect MAPK-JAK/STAT signaling and may be associated with the regulation of the cell cycle or chemoresistance (82). Similarly, changes in *Propionibacterium acnes* abundance may relate to fatty acid metabolism-related pathways (83, 84). Alterations in taxa such as *Methylobacterium*, *Propionicimonas*, and *Caulobacteraceae* have been linked to modulation of PI3K/AKT, MAPK, and Wnt/β-catenin signaling pathways.

**Table 1:** Intratumoral bacterial genera and their roles in breast cancer progression.

Genus	Association with progression	Proposed mechanism	Evidence level and models	References
*Fusobacterium*	Pro-tumor potential	Fap2-GalNAc → tumor colonization; FadA → Wnt/β-catenin↑ + NF-κB↑ + E-cadherin↓ + T-cell function↓ + immunosuppressive microenvironment → tumor progression.	Level 1	(31, 54–57)
*Streptococcus*	Pro-tumor potential	NF-κB↑ + platelet aggregation pathways↑ → NETs↑ + fibrin clots↑ →pro-inflammatory microenvironment → tumor metastasis.	Level 2	(58)
*Pseudomonas*	Pro-tumor potential	TGF-β + PI3K/Akt + MAPK signaling pathways↑ → ErbB2 phosphorylation + downstream transcription factors↑ → cell apoptosis↓ + tumor progression↑ + chemoresistance↑.	Level 1	(59–62)
*Bacteroides*	Pro-tumor potential	BFT-1 toxin → NOTCH1-HEY1 pathway↑ → NUMB degradation → breast cancer stem cell↑ → Tumor progression↑ + chemoresistance↑.	Level 1	(63, 64)
*Prevotella*	Pro-tumor potential	IPyA↓ → UHRF1↑ + AMPK pathway↓ → tumor progression↑.	Level 1	(65)
*Staphylococcus*	*S. epidermidis*: pro-tumor potential	TLR2/7↑ → inflammatory factors↑ → Treg↑ → antitumor immunity↓ + secretes toxins ↑+ ROS↑ → DNA damage.	Level 1	(32, 66–68)
	*S. aureus*: exerts antitumor or pro-tumor potential in a context-dependent manner	1. Anticancer: RLR pathway ↑(TNBC) → STAT3↓ → CD8+ T-cell↑ → tumor progression↓. 2. Pro-cancer: (specific conditions) NF-κB↑ + CCL5-CCR5 axis↑ → DNA damage↑ (without cell apoptosis) → cell cycle progression + cell migration + invasion↑.		
*Methylobacterium*	Antitumor potential	TLR/NF-κB ↓inflammatory pathway → carotenoid metabolic pathway↑ + adaptive immune system → tumor progression↓.	Level 2	(69, 70)
*Sphingomonas*	Antitumor potential	iNKT↑ → Treg↓ → antitumor immunity↑.	Level 3	(71, 72)
*Bifidobacterium*	Antitumor potential	SMAD4/TGF-βpathways↑ → tumor-suppressor gene↑ → EMT↓ → tumor cell proliferation and metastasis↑.	Level 2	(73–77)
*Lactobacillus*	Antitumor potential	AhR pathway↑ → tumor cell proliferation↓ → apoptosis↑ → immune surveillance↑.	Level 2	(78–81)

↑, upregulation/activation; ↓, downregulation/inhibition; →, promote/induce/lead to; +, simultaneous occurrence/synergistic effect.

Evidence classification: level 1: evidence derived from human breast cancer (BC) clinical cohorts combined with direct mechanistic validation in BC-specific experimental models; level 2: findings primarily based on BC-related animal or cell models; human clinical relevance or causality remains to be established; level 3: observations based solely on correlations in human cohorts or biological hypotheses extrapolated from non-BC systems.

Mechanisms described are based on current research trajectories and should be interpreted as potential associations rather than established causal drivers in clinical breast cancer.

From a metabolic interaction perspective, bidirectional relationships have been proposed between tumor cells and ITM. For example, under hypoxic conditions, tumor cells secrete lactate and release metabolites such as glutamine, which may provide nutrients for facultative anaerobes in the TME (85, 86). Some microbiota also produce indole derivatives, short-chain fatty acids (e.g., butyrate), and other amino acid-derived metabolites, which may influence host cell metabolism and signaling pathways (87). Such metabolic interactions could form feedback loops associated with tumor cell adaptation to hypoxia and nutrient deprivation (88, 89). The relationship between microbiota and treatment response has also been explored. Certain microbiota taxa may regulate drug-metabolizing enzymes, such as CYP3A4, or efflux pumps, including ABCB1, potentially affecting intracellular concentrations of chemotherapeutic agents such as paclitaxel and doxorubicin (90). In addition, biofilm formation may further impede drug penetration (91), whereas microbe-derived antioxidant enzymes, such as catalase, may modulate drug penetration and oxidative stress, potentially contributing to chemoresistance (92, 93). Exosome-mediated signaling has been proposed as another mechanism of tumor–microbe interaction (94), in which microbe-derived exosomes carrying small RNAs, lipopolysaccharides (LPS), or metabolic enzymes can enter host cells and modulate their signaling networks (95–97), while exosomes from tumor cells may reciprocally influence the local microbiota community.

Overall, current evidence indicates that ITM may be associated with breast cancer progression through modulation of signaling pathways, metabolic interactions, and treatment responses. However, most findings are derived from correlative or preclinical studies and are insufficient to establish ITM as definitive drivers of breast cancer development. Future research incorporating rigorous methodological controls and prospective human studies is needed to clarify their causal roles and clinical significance of ITM.

### 3.4 Intratumoral microbiota and breast cancer metastasis

Distant metastasis is a key determinant of prognosis in breast cancer. ITM may be associated with key steps of metastasis, including EMT, CTC survival, and niche formation. These associations are primarily observed in preclinical or *in vitro* studies, and their direct contribution to human metastasis remains unproven.

Mechanistic studies in animal models or *in vitro* systems suggest that certain bacteria may interact with CTCs, potentially enhancing their survival under mechanical stress (98). For example, Fusobacterium nucleatum may influence the Rho-GTPase pathway (99, 100), F-actin organization, and RhoA/β-catenin signaling pathways, which are linked to cell migration and invasion (101, 102). Similarly, adhesins such as FadA can bind E-cadherin and activate the β-catenin/CHK2 axis, potentially inducing EMT (103–105). These effects remain to be validated in human tumors.

Meanwhile, microbiota metabolites may modulate signaling pathways such as TLR4/NF-κB and STAT3, and influence regulatory T cells (Tregs) and myeloid-derived suppressor cells (MDSCs) (106–108), contributing to an immunosuppressive TME (109). Cancer-associated fibroblasts (CAFs) may also be influenced by tumor-secreted factors and microbe-associated signals, potentially affecting ECM remodeling, vascular permeability, and immune cell recruitment (110, 111). Transcriptomic analyses indicate compositional shifts in metastatic breast cancer, such as increased *Proteobacteria* and *Firmicutes* and decreased *Actinobacteria*, which correlate with invasive phenotypes. These observations are largely correlative, and the functional significance remains uncertain (11, 112, 113).

Other microbiota components, including fungi such as Malassezia globosa, have been linked to increased expression of immunosuppressive cytokines, although their role in metastasis is unclear (114). Overall, ITM may be involved in processes relevant to metastatic potential, immune regulation, and treatment responses, but these effects are largely speculative and derived from preclinical studies.

## 4 Immune regulation by intratumoral microbiota

ITM may regulate the tumor immune microenvironment through multiple pathways. Most evidence is derived from *in vitro* studies, animal models, or correlative analyses, and causal roles in human breast cancer remain unproven. This section summarizes potential ITM-mediated effects on innate and adaptive immunity, with explicit reference to the type of evidence.

### 4.1 Innate immune activation and modulation

Microbiota within the breast cancer TME may modulate innate immunity through interactions with host immune cells. ITM is typically recognized by host PRRs, which may influence innate immune signaling. Among PRRs, Toll-like receptors (TLRs) play a central role (115–117). They are primarily expressed on immune cells and detect various microbe-associated molecular patterns (MAMPs), initiating early immune responses (118, 119). For example, TLR4 recognizes lipopolysaccharide (LPS), TLR2 detects peptidoglycan and lipoteichoic acid, and TLR5 binds bacterial flagellin (120–123). NOD-like receptors (NLRs), localized in the cytoplasm, can detect intracellular bacterial components after ITM entry into immune or tumor cells, potentially affecting intracellular signaling (124–126). NOD1 recognizes diaminopimelic acid, and NOD2 targets muramyl dipeptide from bacterial peptidoglycan. RIG-I-like receptors (RLRs) primarily sense intracellular viral RNA but may also recognize bacterial nucleic acids (127–130). Cytosolic DNA sensors, such as cGAS–STING, are also involved (131, 132).

At the level of innate immunity, tumor-associated macrophages (TAMs) are among the most responsive immune cells to signals from the microbiota. Preclinical studies suggest that microbiota signals may influence TAM polarization, leading to a phenotype characterized by increased IL-10, TGF-β, and VEGF (133). DCs and neutrophils may also be affected (134). Bacterial signals can induce type I interferon responses; sustained stimulation may lead to DC tolerance, including downregulation of MHC-I and co-stimulatory molecules, and impaired antigen presentation (135–137). Additionally, neutrophils may form neutrophil extracellular traps (NETs) in response to microbiota signals (138–140).

**Figure 2:**
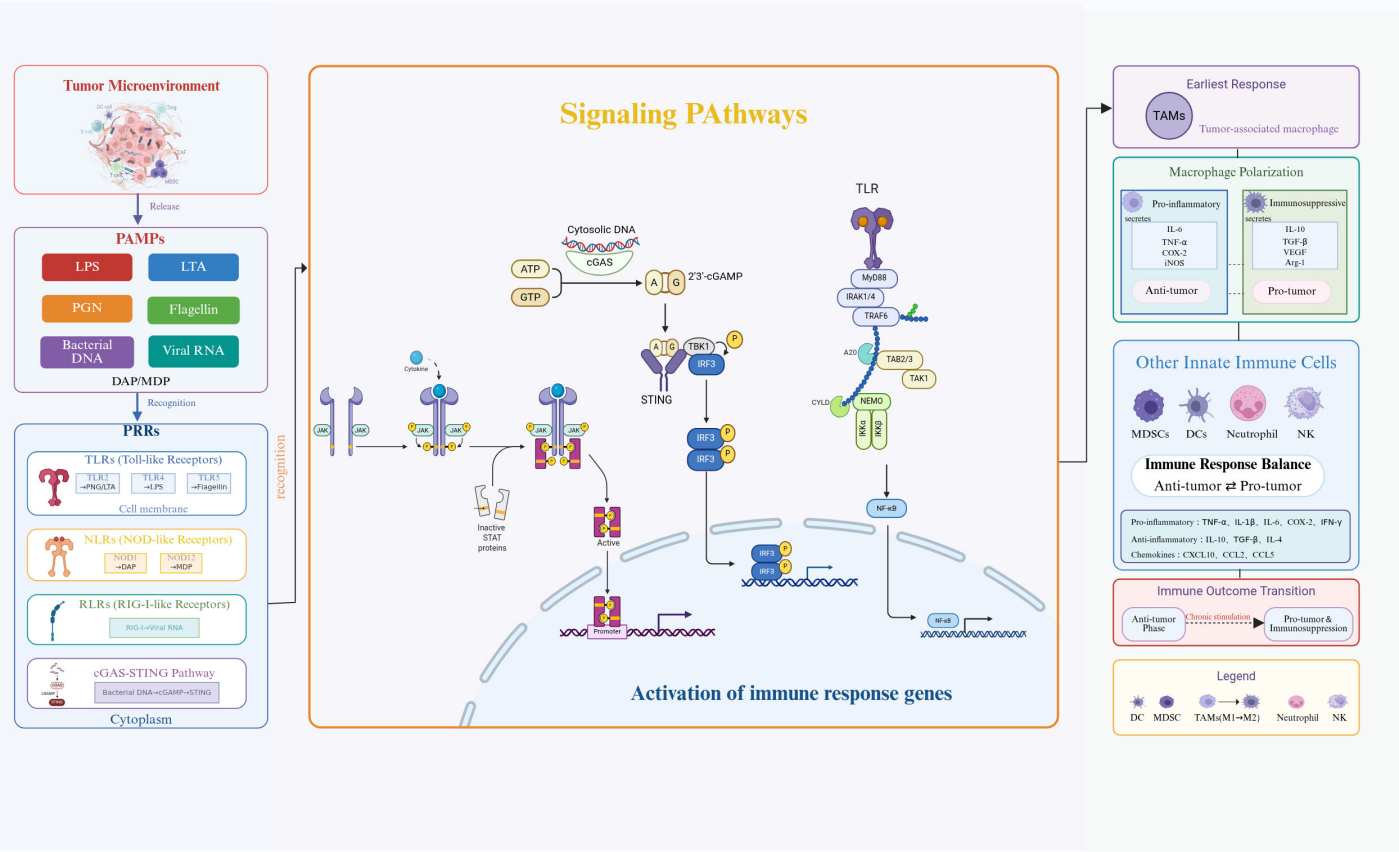
Intratumoral microbiota and innate immune signaling pathways. This figure illustrates the activation of innate immune signaling pathways by ITM-derived microbe-associated molecular patterns (PAMPs) within the tumor microenvironment (TME). To ensure rigorous interpretation, the diagram is stratified by evidence levels: the first two background-colored sections depict signaling cascades (e.g., TLR, cGAS-STING, and NF-κB/STAT3 pathways) and macrophage polarization supported by direct preclinical or *in vitro* evidence. In contrast, the third section highlights inferred or conceptual mechanisms regarding chronic stimulation and long-term immune outcome transitions that have yet to be fully validated in human breast cancer systems. Solid arrows indicate the direction of signaling transduction or molecular interactions. An angled arrow indicates activation of signaling pathways or induction of gene transcription by transcription factors. “P” denotes phosphorylation. Created in https://BioRender.com.

Overall, ITM may be associated with innate immune regulation via PRR-mediated signaling pathways and may contribute to inflammatory and immunosuppressive states. Causal roles in human breast cancer are not established, and further studies are required to validate these associations. (**Figure 2**).

### 4.2 Adaptive immune regulation

At the level of adaptive immunity, preclinical and correlative studies suggest that sustained microbiota-associated signaling may contribute to immunosuppressive phenotypes, including increased Tregs and reduced cytotoxic T-cell activity, potentially accompanied by upregulation of immunosuppressive factors and reduced activity of CD8^+^ cytotoxic T lymphocytes (CTLs) (141). Persistent activation of PRR-related signaling has been observed in some models to correlate with PD-L1 expression, which may contribute to T-cell functional restriction via the PD-1/PD-L1 pathway (142).

In metastatic breast cancer clinical samples, the proportion of PD-1-high CD8^+^ T cells has been reported to correlate with the abundance of *Proteobacteria*, suggesting that ITM composition may be linked to T-cell exhaustion (143). Beyond T cells, interactions between stromal and immune cells, such as cancer-associated fibroblasts (CAFs), may influence immune cell recruitment and the local metabolic environment, potentially contributing to an immunosuppressive state (144–147). Bacterial signal-enriched regions have been observed to co-localize with elevated expression of ARG1, IDO1, and PD-L1 in CAFs and TAMs, reflecting spatial co-occurrence rather than confirmed causality (148).

Furthermore, some studies also suggest that sustained microbiota stimulation may affect DC maturation and antigen presentation, potentially influencing T-cell responses. Collectively, these observations indicate a trend toward an immunosuppressive TME. However, these findings remain largely correlative, and causal roles in human breast cancer are not established.

### 4.3 Regulatory influence of intratumoral microbiota on immune checkpoints

Immune checkpoint pathways are critical for maintaining immune homeostasis and regulating antitumor responses, and their aberrant activation is often associated with T-cell functional exhaustion. Some studies have explored the potential relationship between ITM and immune checkpoint expression (149).

From a spatial perspective, transcriptomics and multi-omics analyses indicate that ITM may be associated with immune checkpoint regulation via inflammatory and metabolic pathways. Spatial analyses further suggest that microbiota signals are co-localized with PD-L1 expression, although causality remains unclear.

Mechanistically, microbe-derived molecules (e.g., LPS) or the cytokines they induce (e.g., IL-6, IL-10, and TGF-β) may influence inflammatory signaling networks, thereby affecting PD-L1 expression. In addition, metabolic processes, including lactate accumulation and tryptophan metabolism, have been proposed to participate in immune checkpoint regulation (150–152). These observations are primarily derived from preclinical or correlative studies.

Beyond the PD-1/PD-L1 axis, other immune checkpoint molecules, including CTLA-4, TIM-3, LAG-3, and TIGIT, may exhibit coordinated expression under chronic inflammatory or immunosuppressive conditions, potentially contributing to T-cell functional exhaustion.

### 4.4 Metabolic regulation by intratumoral microbiota

Beyond inflammatory signaling, microbe-associated metabolic processes may modulate the tumor immune microenvironment. Preclinical studies suggest that ITM and their metabolic products could influence immune cell functional states and immune checkpoint expression by affecting metabolic homeostasis and signaling pathways.

Short-chain fatty acids (SCFAs), such as butyrate and propionate, have been observed in preclinical models to influence chromatin accessibility via histone deacetylase (HDAC) regulation, potentially affecting the expression of immune-related genes. SCFAs may also signal through G protein-coupled receptors (e.g., GPR43), potentially participating in immune regulation. Lactate accumulation within the TME reflects metabolic reprogramming and may alter local pH, potentially impacting effector T-cell activity and enhancing immunoregulatory phenotypes of myeloid cells under preclinical conditions. Hypoxia and metabolic stress, such as those associated with HIF-1α, have similarly been linked to immunosuppressive phenotypes in preclinical studies. The tryptophan metabolic pathway is another potential regulator of tumor immunity. Conversion of tryptophan to kynurenine may activate the aryl hydrocarbon receptor (AhR) signaling pathway, potentially increasing the expression of immunosuppressive genes. In addition, microbe-derived indole metabolites could influence immune cell functional states. Alterations in arginine metabolism may also play a role; in preclinical models, arginine depletion can limit T-cell metabolism and alter immune checkpoint expression, whereas restoring arginine levels can improve T-cell function and enhance immune responses. Collectively, these observations suggest that metabolic states may have bidirectional effects on immune regulation, based primarily on preclinical or correlative studies.

Overall, microbe-associated metabolic products may dynamically regulate the tumor immune microenvironment by influencing chromatin, cellular metabolism, and signaling pathways. These processes may complement inflammatory signaling and immune cell interactions, providing a more comprehensive framework for understanding potential immunomodulatory effects, whereas causal roles in human breast cancer remain to be established.

### 4.5 Dual nature of immune regulation

The effects of ITM on the immune system appear to be context-dependent and potentially bidirectional. Under certain preclinical conditions, microbe-associated signals have been linked to enhanced antitumor immunity (153, 154), partly through activation of innate immune pathways and induction of type I interferon (IFN-I), which may promote DC maturation and natural killer (NK) cell activation, thereby supporting CD8^+^ T-cell responses. In breast cancer animal models, the TLR3 agonist poly(I:C) has been shown in breast cancer models to increase T-cell infiltration and cytokine production, contributing to tumor growth inhibition when combined with other interventions. Conversely, immune outcomes are strongly influenced by the TME. Hypoxia, metabolic stress, and host-related factors such as age may interact with inflammatory signaling, shaping immune cell functional states and favoring immunosuppressive phenotypes under certain conditions (155–157).

Microbiota composition and diversity may further modulate these effects. Higher microbial diversity has been associated with enhanced immune activity, whereas dysbiosis is more frequently linked to immunosuppressive states (158–160). Interactions among microbial taxa and their metabolic products may collectively influence the directionality of immune responses (66).

Overall, ITM-associated immune effects are dynamic and context-dependent, shaped by signal strength, microenvironmental conditions, and host immune status. However, these observations are largely based on preclinical or correlative evidence, and causal roles in human breast cancer remain unproven.

## 5 Microbiome-based therapeutic strategies

**Figure 3:**
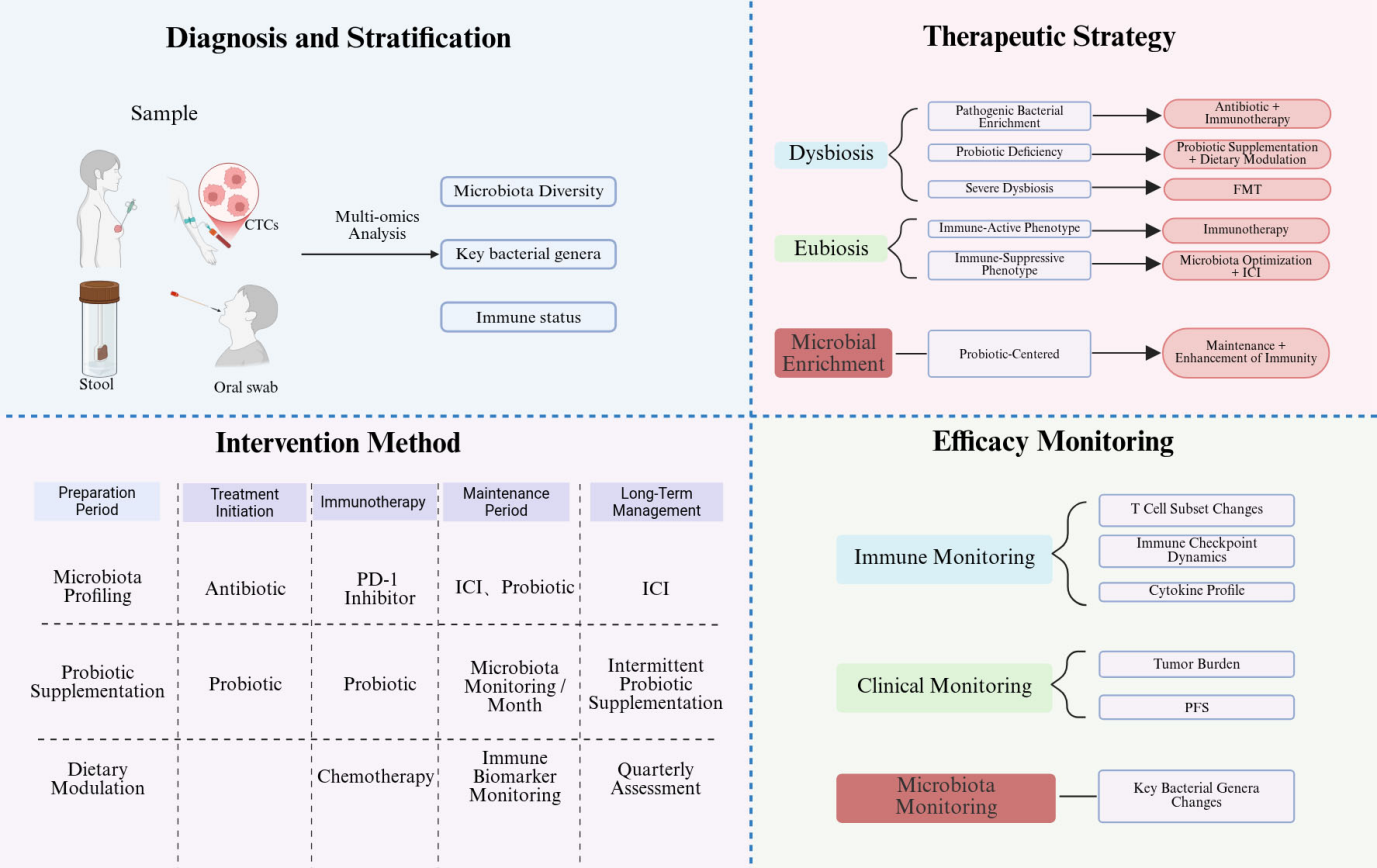
Diagnosis, stratification, and microbiome-based therapeutic strategies. This multiaxial framework organizes potential clinical applications into four domains based on their translational readiness. Diagnosis and stratification methods utilizing multi-omics analysis are largely supported by human observational studies. However, the proposed therapeutic strategies for managing dysbiosis (e.g., FMT and dietary modulation) and the long-term intervention protocols remain primarily hypothesis-driven or based on early-phase experimental evidence from preclinical models and other tumor types. These models serve as a guide for future clinical trials rather than established protocols for current clinical practice. Created in https://BioRender.com.

Current evidence primarily supports a correlative relationship between ITM composition and breast cancer metastasis. Although preclinical models suggest that specific taxa may influence metastatic phenotypes, their causal roles in human cohorts remain unproven (**Figure 3**).

### 5.1 Microbiome intervention approaches

Current microbiota-targeted strategies include probiotic or synbiotic supplementation, precise antibiotic administration, and fecal microbiota transplantation (FMT). Preclinical and early-phase studies have explored their potential mechanisms, but clinical feasibility in breast cancer remains unproven (161, 162). Probiotics and prebiotics, such as *Lactobacillus* and *Bifidobacterium* (73), have been associated with improved barrier function and immune modulation in experimental models. However, clinical evidence in breast cancer is limited, and variability in microbiota composition and the lack of standardized protocols constrain their applicability. Antibiotics may alter tumor-associated microbiota and influence immune or metabolic states. Broad-spectrum antibiotics can induce dysbiosis, which has been linked to reduced immunotherapy responses in some studies. However, clinical validation in breast cancer is lacking, and precise administration (narrow-spectrum, short-course, targeted) with monitoring of microbiota dynamics is recommended if applied experimentally.

FMT has been shown to modulate host immunity in animal models and other tumor types, but evidence in breast cancer remains scarce (163, 164). Clinical use requires strict safety considerations, including donor screening and pathogen testing (165, 166). Overall, these strategies remain experimental, and their efficacy and safety in breast cancer require further validation.

### 5.2 Microbiome and immunotherapy

Immune checkpoint inhibitors (ICIs) represent a major advance in cancer immunotherapy; however, their efficacy in breast cancer, particularly triple-negative breast cancer (TNBC), remains limited (167). Compared with melanoma and non-small cell lung cancer, TNBC typically exhibits lower response rates, potentially due to reduced immune infiltration and increased immunosuppressive features (168). Preclinical and correlative studies suggest that the microbiome, including gut microbiota and ITM, may modulate antitumor immunity. Microbe-associated metabolites have been linked to changes in immune checkpoint expression and immune cell function. However, the impact of microbiota-modulating strategies, such as antibiotic use, on ICI responses remains inconsistent and incompletely understood (169).

Preclinical studies further indicate that microbiota-targeted interventions, including probiotics, antibiotics, and fecal microbiota transplantation (FMT), may influence the tumor immune microenvironment and responses to immunotherapy. In contrast, broad-spectrum antibiotics may disrupt microbial homeostasis and have been associated with reduced therapeutic efficacy in some models. FMT has shown immunomodulatory effects in animal studies, but its relevance to breast cancer remains unclear. Overall, these findings are primarily preclinical, and their clinical significance has yet to be established.

### 5.3 Clinical evidence and future applications

Current clinical studies are mostly descriptive, with limited interventional data. Some correlation analyses suggest that ITM composition in breast cancer patients may be associated with immune infiltration or prognostic indicators. For example, higher abundance of Lactobacillus and Bifidobacterium has been linked to enhanced immune signatures, whereas enrichment of Fusobacterium is associated with immunosuppressive features and a poorer prognosis (54, 170–172). However, prospective and adequately powered clinical trials are lacking. Consequently, the potential of microbiota-targeted interventions to enhance immunotherapy efficacy or prevent metastasis in breast cancer remains speculative. At present, such approaches should be considered hypothesis-generating, with evidence interpreted according to its level (preclinical, indirect, or early clinical).

### 5.4 Challenges and limitations

Current research on microbiota-targeted interventions holds translational potential, but several challenges remain (173). First, safety and interindividual variability are major concerns, particularly given the complexity of treatment regimens in breast cancer. Modulation of the microbiota may introduce risks such as infection, metabolic disturbances, or immune-related adverse events. Second, baseline heterogeneity in microbiota composition, immune status, and lifestyle factors may influence treatment outcomes. Third, lack of technical standardization limits comparability across studies, particularly in low-biomass settings where contamination control is critical. In addition, microbiota composition exhibits spatial and temporal variability, and single-point sampling may not adequately capture these dynamics. The absence of longitudinal data further limits the ability to establish causal relationships between microbiota changes and immune responses.

In summary, ITM remains an exploratory target and a potential biomarker in breast cancer immunotherapy. Future research should prioritize large-scale, multicenter studies and integrate multi-omics approaches to improve mechanistic understanding and predictive modeling. Standardized methodologies and longitudinal study designs will be essential to determining whether microbiota modulation can contribute to metastasis prevention or immunotherapy sensitization. However, causal roles and clinical benefits in breast cancer remain unproven.

## 6 Future directions and research challenges

### 6.1 Key scientific questions

The biological significance of intratumoral microbiota (ITM) in breast cancer metastasis is increasingly recognized. ITM, typically low in biomass and predominantly intracellular, may be associated with tumor cell survival during dissemination and modulation of the metastatic niche through immune regulation (174). However, current evidence remains largely preclinical and correlative, and the causal chain linking specific microbiota taxa, signaling pathways, and disease stages to immune outcomes remains poorly defined.

Key questions in immune regulation can be summarized in three interrelated dimensions. First is origin, remodeling, and organ specificity: ITM may derive from multiple sources, including the gut, skin, and mammary ducts, and may be selectively shaped by organ-specific microenvironments during metastasis. However, longitudinal human studies with multiorgan sampling remain limited (173, 175).

Second is mechanistic mapping of receptor–pathway–phenotype relationships: It remains unclear how PRR networks respond to ITM-derived signals and how these responses translate into defined immune phenotypes (171, 176–178). Evidence linking microbiota components to immune activation and metastatic outcomes remains incomplete.

Third is adaptive immune regulation: High-resolution studies are scarce, and it remains uncertain how specific microbiota taxa influence immune cell populations or whether they affect micrometastatic clearance and immune evasion (179).

These mechanistic gaps translate into challenges for clinical development. Distinguishing whether ITM act as predictive biomarkers or causal drivers of immunotherapy response remains difficult. In addition, systemic microbiota-targeted interventions may disrupt host microbiota balance and impair antitumor immunity (180–183).

Methodologically, reliable detection of ITM requires rigorous contamination control and standardized workflows, supported by orthogonal validation approaches, including spatial omics and imaging-based techniques (184, 185). Overall, there is a need to develop integrated, spatially resolved maps of microbe–host interactions and to validate their clinical relevance in well-controlled prospective studies.

### 6.2 Future technical development directions

In recent years, advances in multi-omics technologies have enabled breast cancer research to move from single-dimensional genetic analyses to integrated studies of cellular ecology and the TME. Single-cell RNA sequencing (scRNA-seq) and spatial multi-omics technologies allow researchers to characterize the composition, functional states, and spatial relationships of different cell types in tumor tissue at high resolution (186).

For instance, scRNA-seq-based breast cancer cell atlases have revealed complex interaction networks among epithelial, stromal, and immune cells, whereas spatial transcriptomics and spatial proteomics technologies (e.g., 10x Visium, IMC, and CODEX) help elucidate the spatial distribution patterns of these cell populations within the tissue microenvironment (82, 187). Consequently, research on the TIME has advanced from mere “compositional analysis” to “spatial functional ecology”. Simultaneously, high-dimensional immune phenotyping methods (e.g., CyTOF, CITE-seq, and multiplexed immune imaging) provide powerful tools for dissecting the tumor immune ecology (188). By integrating information such as surface proteins, TCR clones, and metabolic features, researchers can more accurately delineate the functional lineages of immune cells and their dynamic changes.

Increasing evidence supports the presence of predominantly intracellular microbiota within tumor tissues, which may be associated with immune status and tumor metabolism. However, methodological challenges related to low biomass necessitate rigorous contamination control and standardized workflows, as highlighted in recent methodological guidelines (189).

Emerging integrative approaches, such as INVADEseq, enable simultaneous profiling of host transcriptomes and bacterial sequences at single-cell resolution (190). These methods provide new opportunities to investigate the spatial organization of microbiota–host interactions. Integration of spatial multi-omics and immune imaging may further enable reconstruction of multicellular interaction networks, offering insights into how ITM may influence the tumor immune microenvironment and metastatic processes (82).

### 6.3 Clinical translation pathway

Future research should focus on developing cross-scale, multimodal analytical frameworks (186–188). For example, integrating spatial transcriptomics, immune profiling, and microbiome analysis in paired primary and metastatic tumor samples may help identify co-occurrence patterns between microbiota signals and immune features. These associations could provide insights into potential mechanisms by which the ITM might interact with immune responses and metastatic processes. Additionally, integrating these molecular features into multimodal predictive models may support hypothesis generation for potential biomarkers of immunotherapy response. However, their clinical applicability remains uncertain, as current evidence is largely preclinical or correlative.

In parallel, microbiota-targeted strategies, including improved detection of low-biomass microbiota and approaches to modulate microbial communities, warrant further investigation. Preclinical studies suggest that such strategies may influence immune activity, but their efficacy and safety in breast cancer patients remain to be established.

Overall, these approaches provide a framework for mechanistic and hypothesis-driven research rather than immediate clinical application.

### 6.4 Necessity of interdisciplinary collaboration

Establishing interdisciplinary collaboration across oncology, immunology, microbiology, and computational sciences is essential for advancing this field (11, 189, 190). Such integration enables coordinated efforts in sample processing, mechanistic investigation, and data analysis, thereby supporting a more robust research pipeline from discovery to validation and facilitating clinical translation.

**Figure 4:**
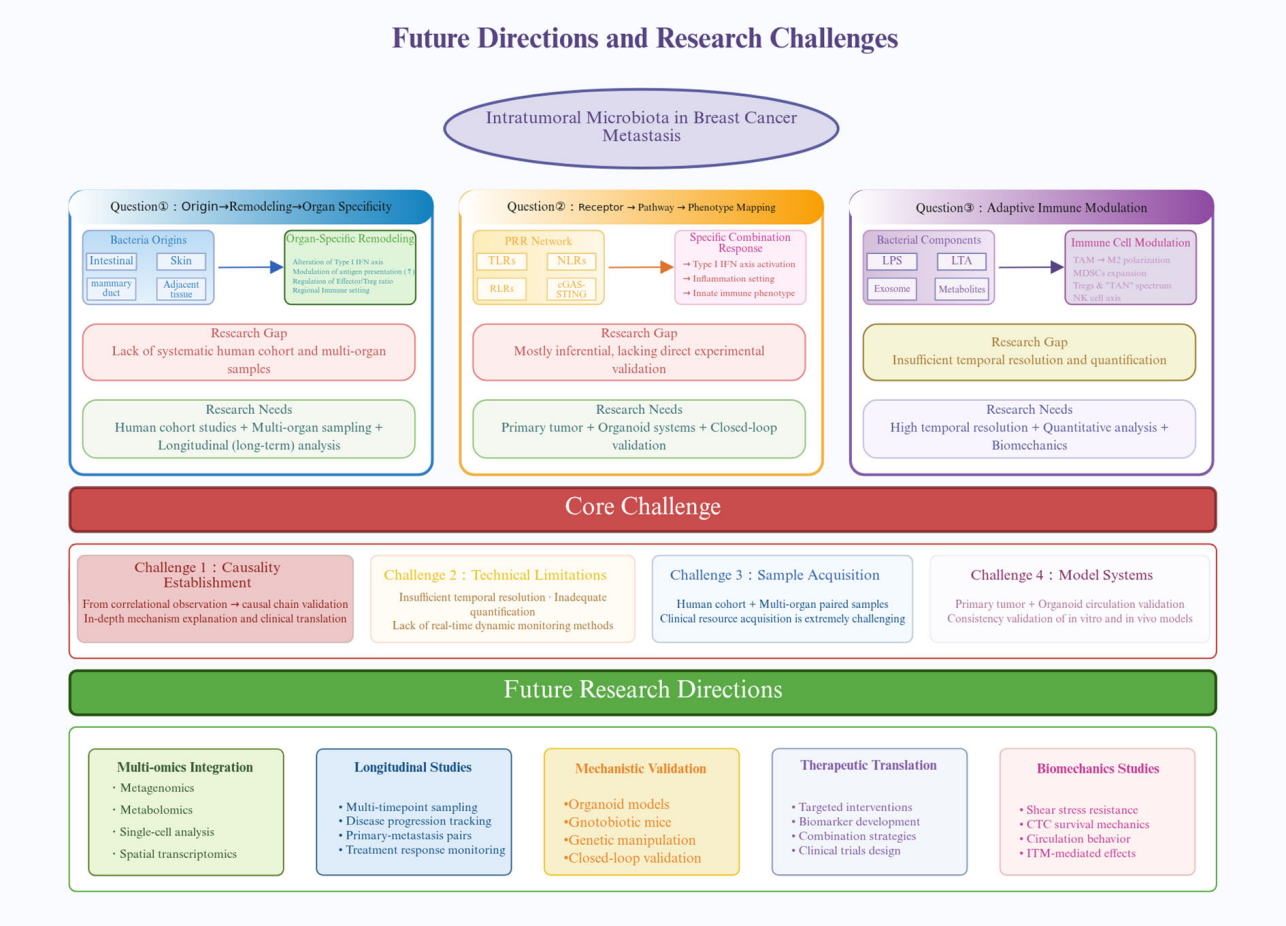
Future directions and research challenges in breast cancer microbiomics. This summary outlines the key scientific questions and core challenges in breast cancer intratumoral research. Key focus areas include bacterial origin/remodeling (Question 1), receptor-phenotype mapping (Question 2), and adaptive immune modulation (Question 3). The framework emphasizes the critical transition from correlational observations to causal validation, highlighting the need for longitudinal human cohorts, multiorgan paired sampling, and standardized model systems to overcome current technical limitations and establish the clinical significance of the ITM in metastasis. Created in https://BioRender.com.

In conclusion, the integration of single-cell and spatial multi-omics, high-dimensional immune profiling, and ITM analysis is driving breast cancer research toward a more unified “microecology–immunity–metastasis” framework. While these advances provide new opportunities to elucidate microbe–host interactions, current evidence remains largely exploratory. Causal roles and clinical applicability have yet to be established, and existing findings should be interpreted primarily as guiding future research directions. (**Figure 4**).

## 7 Conclusion

In summary, immune regulation during breast cancer metastasis involves complex interactions within the TME. ITM may be associated with immune evasion and tumor progression; however, its precise role in human breast cancer remains unclear. Current evidence, largely derived from preclinical and correlative studies, suggests that microbe-associated signals may influence immunosuppressive phenotypes and metastatic processes through the modulation of immune cell function, inflammatory signaling, and metabolism. Future research should prioritize rigorous validation using standardized methodologies, longitudinal human cohorts, and integrated multi-omics approaches. Although emerging technologies, including single-cell and spatial multi-omics, provide new opportunities to investigate microbe–host interactions, their clinical relevance remains to be established. Overall, advancing this field will require careful methodological design, evidence integration, and critical evaluation.
